# The use of thermostable fluorescent proteins for live imaging in *Sulfolobus acidocaldarius*

**DOI:** 10.3389/fmicb.2024.1445186

**Published:** 2024-09-09

**Authors:** Alejandra Recalde, Jasmin Abdul-Nabi, Pierre Junker, Chris van der Does, Jana Elsässer, Marleen van Wolferen, Sonja-Verena Albers

**Affiliations:** Molecular Biology of Archaea, Faculty of Biology, University of Freiburg, Freiburg im Breisgau, Germany

**Keywords:** *Sulfolobus*, fluorescent microscopy, thermal fluorescent protein, archaea, *in vivo* localization, thermomicroscopy

## Abstract

**Introduction:**

Among hyperthermophilic organisms, *in vivo* protein localization is challenging due to the high growth temperatures that can disrupt proper folding and function of mostly mesophilic-derived fluorescent proteins. While protein localization in the thermophilic model archaeon *S. acidocaldarius* has been achieved using antibodies with fluorescent probes in fixed cells, the use of thermostable fluorescent proteins for live imaging in thermophilic archaea has so far been unsuccessful. Given the significance of live protein localization in the field of archaeal cell biology, we aimed to identify fluorescent proteins for use in *S. acidocaldarius*.

**Methods:**

We expressed various previously published and optimized thermostable fluorescent proteins along with fusion proteins of interest and analyzed the cells using flow cytometry and (thermo-) fluorescent microscopy.

**Results:**

Of the tested proteins, thermal green protein (TGP) exhibited the brightest fluorescence when expressed in *Sulfolobus* cells. By optimizing the linker between TGP and a protein of interest, we could additionally successfully fuse proteins with minimal loss of fluorescence. TGP-CdvB and TGP-PCNA1 fusions displayed localization patterns consistent with previous immunolocalization experiments.

**Discussion:**

These initial results in live protein localization in *S. acidocaldarius* at high temperatures, combined with recent advancements in thermomicroscopy, open new avenues in the field of archaeal cell biology. This progress finally enables localization experiments in thermophilic archaea, which have so far been limited to mesophilic organisms.

## Introduction

Since its discovery in 1962, the green fluorescent protein (GFP) from *Aequorea victoria* and its derivatives have proven to be invaluable tools for observing microscopic life (Pakhomov and Martynov, 2008; Phillips, 2001; Shimomura et al., 1962). When fused to another protein, fluorescent proteins allow the intracellular localization of these and the visualization of processes in living cells, such as cell division (Brzozowski et al., 2019; Stricker et al., 2002), transcription and translation (Cai et al., 2006; Golding et al., 2005), and DNA segregation (Gitai et al., 2005), among others (Meyer and Dworkin, 2007; Phillips, 2001). In archaea, the use of fluorescent proteins has proven to be challenging because of the extreme growth conditions that many of the model organisms live in van Wolferen et al. (2022).

To date, the use of FP in archaea has been limited to mesophilic organisms such as *Haloferax volcanii*. In this organism, specific point mutations in GFP resulted in a protein that is stable under high salinity conditions (Reuter and Maupin-Furlow, 2004) and has successfully been used to study various cellular processes in living cells, including cell division, S-layer synthesis, DNA replication, cell motility, and cell shape (Bisson-Filho et al., 2018; Ithurbide et al., 2022, 2024). Additionally, the creation of an autofluorescence-free *H. volcanii* strain enabled the establishment of live single-molecule microscopy (Turkowyd et al., 2020).

The use of fluorescent proteins in methanogens has been restricted by the anoxic conditions these organisms require, as oxygen is necessary for the proper maturation of the fluorophore (Heim et al., 1994). However, the development of oxygen-independent fluorescence-activating and absorption-shifting tag (FAST) has proven useful for live cell imaging (Hernandez and Costa, 2022). FAST tags exhibit fluorescence upon binding to specific fluorogens. Various FAST variants, with affinity to different ligands, are commercially available (Tebo et al., 2021).

For thermophiles, such as members of the order Sulfolobales, the lack of fluorescent proteins that function at high temperatures has necessitated the use of immunolocalization on fixed cells to visualize cell division proteins (Cezanne et al., 2023; Lindås et al., 2008; Samson et al., 2008; Tarrason Risa et al., 2020). In studies on the thermophilic bacterium *Thermus thermophiles*, superfolder GFP (sfGFP), a variant of GFP was used at 70°C to localize GroES in living cells (Cava et al., 2008). sfGFP was generated to have a high chemical stability and to fold efficiently even in the presence of misfolded fusion proteins (Pédelacq et al., 2006). So far, it was not expressed in any thermophilic Archaea, in this study we therefore tested several presumed thermostable fluorescent proteins in *S. acidocaldarius*.

The thermostable eCGP123 was the first fluorescent protein that was successfully expressed in *Sulfolobus acidocaldarius*, allowing the visualization of biofilms (Henche et al., 2012). It was developed through direct evolution of a Consensus Green Protein (CGP) (Kiss et al., 2009). Further engineering resulted in the so-called thermal green protein (TGP), which is more stable and less prone to aggregation (Close et al., 2015). TGP retained fluorescence *in vitro* after prolonged incubation at 90°C, but it has not been tested in thermophilic organisms *in vivo* so far (Close et al., 2015). Recently, point mutations in TGP led to the development of an enhanced version (TGP-E) (Anderson et al., 2023).

Fluorescent proteins with different colors enable simultaneous labeling of various structures within the cell. In addition to green fluorescent proteins, we therefore tested a few thermo-optimized yellow fluorescent proteins. Mutagenesis of sfGFP and TGP, respectively, led to the development of Superfolder YFP (sfYFP) (Pédelacq et al., 2006), as well as the yellow thermo protein (YTP) and its enhanced version (YTP-E) (Anderson et al., 2023). Moreover, the yellow hyperfolder YFP (hfYFP), derived from mGreenLantern, and its monomeric version mfYFP are highly thermostable, resistant to acidic conditions and to chemical agents used to fixed cells, such as PFA and OsO_4_ (Campbell et al., 2022).

In recent years, there has been growing interest in the cell biology of archaea. Among halophiles, significant developments in cell biology, including the use of diverse fluorescent proteins, have facilitated live microscopy of intracellular structures, comparable to that in bacteria (Bisson-Filho et al., 2018; Ithurbide et al., 2024; van Wolferen et al., 2022). Additionally, the optimization of thermomicroscopy has allowed the visualization of thermophilic organisms, such as the model organism *S. acidocaldarius*, under their natural growth temperatures (Charles-Orszag et al., 2021; Mora et al., 2014; Pulschen et al., 2020). Nevertheless, especially amongst thermophiles, the field of cell biology is still lagging behind, mainly due to the absence of functional thermostable fluorescent proteins. In this study, we therefore explore the use of TGP and other green and yellow thermostable fluorescent proteins in *S. acidocaldarius*. Fusion proteins of TGP with CdvB and PCNA1 confirmed their previously published localization. In addition, in-gel fluorescence of the proteins enabled straightforward detection without the requirement for Western blotting.

## Results

### Finding a suitable fluorescent protein at high temperatures

**FIGURE 1:**
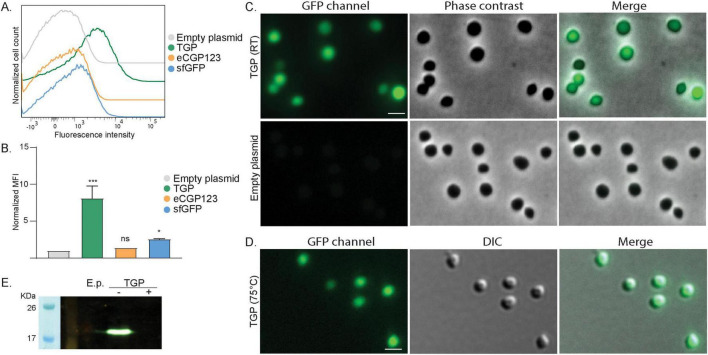
Expression of different green fluorescent proteins in *S. acidocaldarius* **(A)** Flow cytometry on *S. acidocaldarius* cells expressing different green fluorescent proteins induced with 0.2% xylose. Total number of events per sample: 100,000. **(B)** Normalized Median Fluorescent Intensity (MFI) from the flow cytometry experiments. Samples were normalized against the MFI value of the empty plasmid. Results are the average of at least three replicas. Welch’s *t*-test: *** indicating *p* ≤ 0.001, *p ≤ 0.05 and ns: not significant. **(C)** Fluorescent microscopy, phase contrast and merged images of *S. acidocaldarius* cells expressing TGP from a plasmid induced with 0.2% xylose, imaged at RT on agarose pads. **(D)** Fluorescent microscopy at high temperature. DIC and merged images of *S. acidocaldarius* cells expressing TGP, imaged at 75°C. Rulers indicate 2 μm. **(E)** In-gel fluorescence of TGP, when samples were boiled (+) or not (–) in SDS containing buffer. E.p., empty plasmid; RT, room temperature.

In our search for thermostable fluorescent proteins suitable for localization experiments in *S. acidocaldarius*, we first tested three previously published green fluorescent proteins: sfGFP (Pédelacq et al., 2006), eCGP123 (Kiss et al., 2009) and TGP (Close et al., 2015; Table 1). Codon optimized genes were first expressed using our previously published FX expression plasmids (van der Kolk et al., 2020) by inducing protein expression with xylose. To measure their brightness, we performed flow cytometry on the expression cultures (Figures 1A, B and Supplementary Figures 1A, B). *S. acidocaldarius* cells generally exhibit some auto fluorescence. When expressing either sfGFP or eCGP123, fluorescence of the cells was only slightly increased compared to cells with an empty plasmid (Figure 1A and Supplementary Figures 1A, B). The brightest fluorescence could be observed for cells expressing TGP. This could be confirmed by analysis of the normalized median fluorescent intensities (MFI), which showed a significant increase in fluorescence only for cells expressing TGP (Figure 1B). To find optimal conditions, we tested two xylose concentrations to induce expression and different temperatures before injecting the cells in the flow cytometer. Induction with 0.2% xylose resulted in slightly stronger fluorescence than induction with 1% xylose (Supplementary Figures 1A, B). All subsequent experiments where therefore performed with 0.2% xylose. Additionally, preheated samples (kept at around 75°C until injection) and cells transported at room temperature did not show any significant difference in fluorescence (Supplementary Figures 1A, B).

**TABLE 1:** Fluorescent proteins used in this study.

	Oligomeric state	Molecular weight (KDa)	Excitation (nm)	Emission (nm)	Reference	FPbase ID (Lambert, 2019)	Fluorescence
sfGFP (superfolder GFP)	Weak dimer	26.8	488	510	Pédelacq et al., 2006	B4SOW	−
eCGP123 (enhanced consensus fluorescent protein)	Monomer	28.5	493	504	Kiss et al., 2009	GZU2E	−
TGP (thermal green protein)	Monomer	25.4	493	507	Close et al., 2015	K782G	++
hfYFP (hyperfolder YFP)	Weak dimer	26.9	514	529	Campbell et al., 2022	YX459	−/+
mfYFP (monomeric hyperfolder YFP)	Monomer	27.0	515	529	Campbell et al., 2022	PF2S1	+
YTP (yellow thermal protein)		25.4	395, 513	522	Anderson et al., 2023	–	−
YTP-E (enhanced yellow thermal protein)		25.4	513	526	Anderson et al., 2023	–	−

In line with our flow cytometry experiments, cells expressing TGP were clearly more fluorescent than cells expressing either sfGFP or eCGP123 when visualized at RT using fluorescence microscopy (Figure 1C and Supplementary Figure 1C). Importantly, similarly bright fluorescence could be observed when visualized at 75°C, making TGP well-suited for live imaging in *S. acidocaldarius* (Figure 1D).

TGP could also be visualized in-gel using our fluorescence imager. For this, whole cell lysates were loaded on an SDS-gel. Samples that were boiled for 10 min did not show any fluorescence, whereas unboiled samples revealed a clear fluorescent band at the expected height of TGP (Figure 1E).

We have thereby successfully used TGP as a fluorescent marker in *S. acidocaldarius*, facilitating flow cytometry, fluorescence microscopy and in-gel fluorescence. Notably, its fluorescence remains similarly bright under the optimal growth conditions of *S. acidocaldarius*, enabling dynamic live imaging.

### Position of the companion protein and use of linkers affected fluorescence of TGP

**FIGURE 2:**
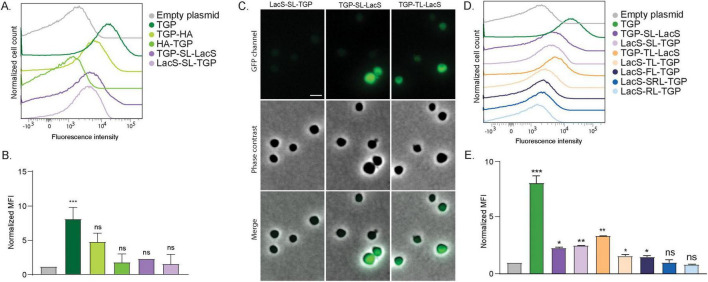
Fluorescence of different TGP fusion proteins. **(A)** Fluorescence intensity of *S. acidocaldarius* cells expressing different TGP-HA and TGP-LacS fusions, as shown with flow cytometry at room temperature. **(B)** Normalized Median Fluorescent Intensity (MFI) from the flow cytometry experiments. Samples were normalized against the empty plasmid MFI value. Results are the average of at least three replicas. **(C)** Fluorescent microscopy, phase contrast and merged images of cells expressing different TGP-LacS fusions. Scale bar: 2 μm. **(D)** Influence of different linkers in the intensity of fluorescence of TGP-LacS fusions as observed in flow cytometry and **(E)** the corresponding MFI values. Samples were normalized against the empty plasmid MFI value. Results are the average of at least three replicas. Number of events for flow cytometry: 100,000. Welch’s *t*-test: *** indicating *p* ≤ 0.001; **p ≤ 0.01; *p ≤ 0.05; ns, not significant; SL, short linker; TL, thermolinker; FL, flexible linker; SRL, semi-rigid linker; RL, rigid linker. For details on the sequences of the linkers, see Table 2.

Because our ultimate goal would be to efficiently localize proteins in *S. acidocaldarius*, we fused TGP both N- and C-terminally to the cytosolic protein LacS (a β-galactosidase from *Saccharolobus solfataricus*, allowing blue/white screening), and an HA tag, linked with a short linker (GGGSGGG, Table 2). Flow cytometry revealed that all fusions resulted in decreased fluorescence when compared to TGP alone, particularly noticeable when TGP was fused at the C-terminus of the protein (Figures 2A, B). The latter could also be observed in fluorescence microscopy (Figure 2C). Because linkers can significantly affect protein activity or even increase expression yields (Waldo et al., 1999; Chen et al., 2013; Li et al., 2016; Ithurbide et al., 2024), we decided to test four different previously published linkers: a linker designed for rapid protein folding assays with fluorescent proteins, which we renamed as thermolinker (Waldo et al., 1999); along with a flexible-, semi-rigid- and rigid linker (Chen et al., 2013; Figures 2D, E). Their sequences are summarized in Table 2.

**TABLE 2:** Sequences of linkers used to fuse TGP to other proteins.

Linker	Sequence	Reference
Short linker	GGGSGGG	This work
Thermolinker	GSAGSAAGSGEF	Waldo et al., 1999
Rigid linker	(EAAAK)5	Li et al., 2016
Flexible linker	(GGGGS)5	Li et al., 2016
Semi-rigid linker	(EAAAK)1(GGGGS)2(EAAAK)2	Li et al., 2016

Fusion proteins linked with the flexible linker (FL), semi-rigid linker (SRL) and the rigid linker (RL) did not exhibited higher fluorescence compared to the fusion with the original short linker (Figures 2D, E). On the other hand, the thermolinker fusion (TL), TGP-TL-LacS, showed an increased fluorescence that was also evident in fluorescence microscopy (Figures 2C–E).

We attempted to enhance brightness and stability of TGP-fusion proteins by introducing point mutations to alter the pKa. Additionally, a cysteine-free version of the TGP was created, considering the rarity of disulfide bonds in archaea. Unfortunately, none of these mutations yielded fluorescence comparable to the original TGP (Supplementary Figure 2). Therefore, we continued using the original TGP in combination with a thermolinker, preferably at the C-terminus of the protein.

### Imaging cell division and replication foci in vivo

Previously, cell division proteins of *Sulfolobus* species have been rewarding targets for localization, providing a clear localization pattern at mid-cell. Especially ESCRT-III homologs CdvB, CdvB1 and CdvB2 form distinct rings. However, due to the absence of thermostable fluorescent proteins, immunolocalization has been the only method to visualize these proteins, involving fixing and permeabilizing cells (Lindås et al., 2008; Pulschen et al., 2020; Samson et al., 2008; Tarrason Risa et al., 2020).

**FIGURE 3:**
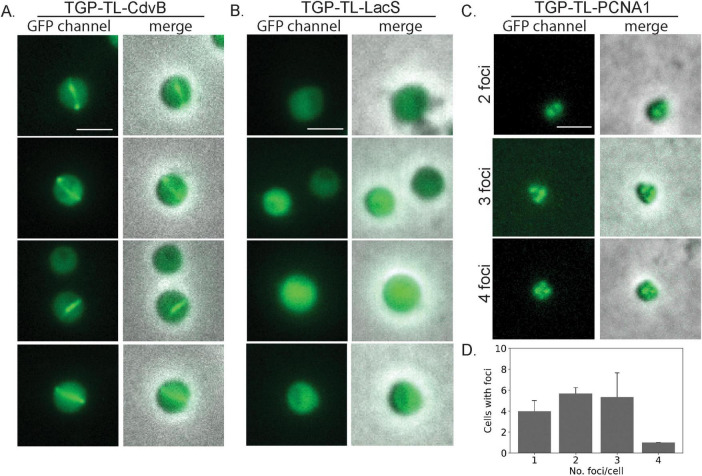
Live cell localization of different TGP fusion proteins. Fluorescence microscopy images and a merge with phase contrast images of **(A)** CdvB rings **(B)** LacS and **(C)** PCNA1. **(D)** Number of foci per cell in PCNA localization (three pictures were used for counting foci, *n* = 48). Scale bar: 2 μm. Additional examples can be found in Supplementary Figure 3.

To test if we could observe similar localization patterns using TGP, we expressed a TGP-TL-CdvB fusion in living *S. acidocaldarius* cells. It was previously shown that inhibiting the proteasome in *S. acidocaldarius* impairs the degradation of CdvB rings (Tarrason Risa et al., 2020), allowing them to be visible for a longer duration. Therefore, we synchronized the cells with acetic acid and subsequently added the proteasome inhibitor bortezomib. As previously observed with immunolocalization, CdvB rings could be observed at midcell after bortezomib arrest (Figure 3A and Supplementary Figure 3), while control cells expressing TGP-TL-LacS showed diffused localization (Figure 3B).

As the second target with a distinct localization pattern, we chose the DNA sliding clamp protein PCNA. In Sulfolobales, three PCNA subunits form a heterotrimeric structure (Acharya et al., 2021; Williams et al., 2006). Immunolocalization of PCNA1 has allowed replisome positioning in *S. acidocaldarius*, revealing one to four foci in the cells during G1 (Gristwood et al., 2012). To test if TGP could similarly be used for PCNA and replisome localization, we expressed TGP-TL-PCNA1 (Saci_0826) under the control of its native promotor. After synchronization, cells were observed under the microscope. Consistent with previous findings (Gristwood et al., 2012), we observed cells with a variable number of foci (between 1 and 4) (Figures 3C, D and Supplementary Figure 3).

We have thereby, for the first time, expressed functional fluorescent fusion proteins *S. acidocaldarius*, enabling the localization of proteins in living cells.

### Other fluorescent proteins

Ideally, we would like to have a diverse color palette of fluorescent proteins for *S. acidocaldarius*, similar to those available for Haloferax (Ithurbide et al., 2024) and Bacteria (Delgadillo-Guevara et al., 2024). To achieve this, we tested a set of previously published thermostable yellow fluorescent proteins (Table 1). These included: YTP, YTP-E, hfYFP and mfYFP. YTP and YTP-E were promising candidates, given the fact that they are derived from TGP (Anderson et al., 2023). Unexpectedly, they exhibited low fluorescence under our tested conditions (Supplementary Figure 4A), even though their expression levels were similar to those of hfYFP and mfYFP (Supplementary Figure 4B).

**FIGURE 4:**
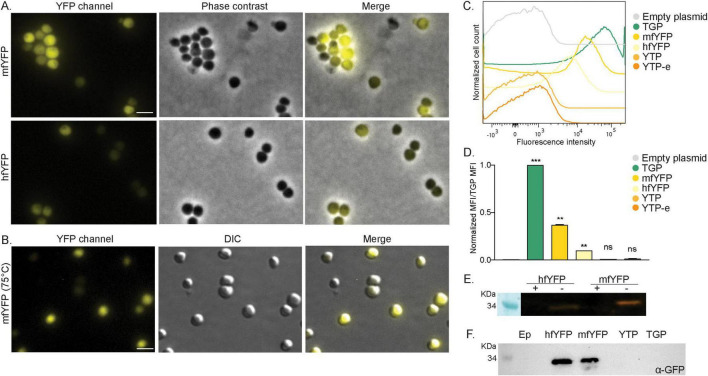
Expression of yellow fluorescent proteins in *S. acidocaldarius*. **(A)** Fluorescent microscopy, phase contrast and merged images of cells on agarose pads at room temperature expressing mfYFP and hfYFP. **(B)** Fluorescent microscopy at high temperature. DIC and merged images of *S. acidocaldarius* cells expressing mfYFP at 75°C using the VAHEAT device. Scale bar: 2 μm. **(C)** Flow cytometry fluorescence intensity of cells expressing different yellow FP at RT and 0.2% xylose and **(D)** normalized Median Fluorescent Intensity (MFI) values from the FC curves. Samples were normalized against the empty plasmid MFI value, and subsequently against the MFI value of TGP. Results are the average of at least three replicas. Welch’s *t*-test: *** indicating *p* ≤ 0.001, **p ≤ 0.01 and ns, not significant. **(E)** In gel fluorescence of hfYFP and mfYFP, when samples were boiled (+) or not (–) in SDS containing buffer. **(F)** Western blot with anti-GFP antibodies.

Two other yellow proteins, hfYFP and its monomeric variant mfYFP, were reported as promising alternatives to enhanced YFP (e-YFP) because of their higher thermostability and the fact that their excitation spectra do not overlap with that of enhanced GFP (e-GFP) (Campbell et al., 2022). Although both hfYFP and mfYFP exhibited lower fluorescence than TGP under our test conditions, fluorescence was still detectable when expressing monomeric mfYFP and imaging at either room temperature or 75°C (Figures 4A, B). Flow cytometry measurements were consistent with our microscopy data (Figure 4C). Both mYFP and hfYFP exhibited significantly higher mean fluorescence intensity (MFI) values compared to the empty plasmid control (Figure 4D). When normalized, the MFI value of mfYFP was found to around 50% of that of TGP (Figure 4D).

Similar to cells expressing TGP, in-gel fluorescence was observed when loading whole cells expressing hfYFP or mfYFP on a gel, though with less intensity (Figure 4E). We also tested the use of commercially available GFP antibodies and were able to detect both hfYFP and mfYFP, but not TGP (Figure 4F). This provides an additional method to detect these proteins without the need for a tag. With mfYFP, we thereby now have an additional thermostable FP with a different excitation wavelength than TGP.

To facilitate cloning in future studies, we created plasmid pSVAara_TGP-TL_FXStop (Supplementary Figure 5A and Supplementary Table 1), based on our previously published set of plasmids (van der Kolk et al., 2020). It harbors the arabinose promoter used in this study, which is responsive to arabinose and xylose (van der Kolk et al., 2020). The plasmid contains *tgp* and the thermolinker. Moreover, *lacI/Z* genes flanked by *Nco*I and *Xho*I allow easy cloning of a gene of interest using blue-white screening. Alternatively, SapI restriction sites would allow for FX-cloning (Geertsma and Dutzler, 2011; van der Kolk et al., 2020). Similarly, plasmids were created for N-terminal and C-terminal tagging with mfYFP pSVAara_mfYFP-TL_FX-Stop and pSVAara_FX_TL-mfYFP-Stop, respectively, (Supplementary Figures 5B, C).

Optimization of these proteins and the search for thermostable fluorescent proteins in other colors will expand our toolbox for live fluorescent thermomicroscopy in *S. acidocaldarius*.

## Discussion

Recent developments in thermomicroscopy significantly contributed to the field of live microscopy of thermophilic archaea, including *S. acidocaldarius*. These advancements have enabled the visualization of dynamic cellular processes in real-time, such as motility and cell division (Charles-Orszag et al., 2021, 2023; Pulschen et al., 2020). With the availability of easy-to-use thermomicroscopy, the development of thermostable fluorescent markers and proteins has become increasingly desirable. While it is possible to utilize dyes for staining components such as DNA or the membrane at high temperatures (Cezanne et al., 2023), the visualization of proteins in Sulfolobales has so far relied on immunostaining (Bisson-Filho et al., 2018; van Wolferen et al., 2022).

In this study, we tested different thermostable fluorescent proteins, linkers, and fusions to identify fluorescent proteins for studying the cell biology of living *S. acidocaldarius* cells. Our findings highlight the successful use of TGP and mfYFP in *S. acidocaldarius* for live imaging. Furthermore, in-gel fluorescence, even under denaturing conditions, allowed easy protein detection on SDS-gels, eliminating the need for western blotting and specific antibodies. When fusing TGP to CdvB and PCNA1, the fusion proteins revealed distinct localization patterns that were previously observed in immunolocalization studies (Gristwood et al., 2012; Samson et al., 2008), demonstrating the effectiveness of TGP in visualizing intracellular proteins. Surprisingly, TGP-based YTP and E-YTP did not exhibit fluorescence despite their thermostability. This could be due to the fact that, although these proteins are more thermostable than the TGP, they have been reported to possess lower quantum yields (a lower proportion of absorbed photons are emitted as fluorescence) (Anderson et al., 2023). We noticed that we obtained highly variable fluorescence between cells when expressing the proteins overnight at higher ODs (data not shown). This could be due to plasmid instability, as it was reported before for bacteria (Wong Ng et al., 2010). Therefore, we generally advise expressing fusion proteins in early exponential phase for maximally 4 h or, ideally, using endogenously tagged proteins. It is worth to notice that the use different linkers can affect the activity of proteins and the fluorescence of FP, and should be explored when constructing fusions (Chen et al., 2013; Li et al., 2016; Ithurbide et al., 2024).

So far, we could only successfully localize proteins that were N-terminally tagged with TGP. Additionally, mfYFP showed significantly less bright fluorescence compared to TGP. Future optimization strategies, such as the use of error-prone PCR libraries, could further enhance the brightness and stability of TGP and mfYFP in *S. acidocaldarius*, as it was done in thermophilic bacteria (Frenzel et al., 2018). The use of brighter and more stable fluorescent proteins will enable us to localize proteins over longer periods using time-lapse microscopy. Moreover, the ongoing search for fluorescent proteins in alternative colors will, in the future, hopefully, enable the co-localization of diverse proteins within the same cell, thereby enhancing our understanding of more complex cellular dynamics and interactions. We anticipate that the utility of TGP and mfYFP can extend beyond *S. acidocaldarius* to other thermophilic archaea, providing valuable insights into protein localization and dynamics within several thermophilic organisms.

## Materials and methods

### Strains, media, transformation and growth conditions

*Sulfolobus acidocaldarius* strain MW001 was grown in Brock medium (Brock et al., 1972) supplemented with 0.1% (*w/v*) N-Z amine (Sigma-Aldrich^®^, Merk KGaA, Burlington, MA, USA), 0.2% (*w/v*) sucrose, and 0.01 mg/mL uracil in the case of uracil autotrophs. Cells were grown in shaking conditions at 120 rpm and 75°C. For expression cultures, 0.2 or 1% of xylose was added during the exponential phase (OD_600_ 0.3–0.5). Subsequently, the cultures were incubated at 75°C for 4 h. For plates, two times concentrated Brock medium supplemented with 6 mM CaCl_2_, 20 mM MgCl_2_, 0.2% N-Z-amine (*w/v*), and 0.4% dextrin (*w/v*) was pre-warmed and mixed in the same volume of freshly boiled 1.4% gelrite (Carl Roth, Karlsruhe, Germany).

Competent cells were prepared as previously described (Wagner et al., 2012; Ye et al., 2022). For transformation, 100–200 ng of methylated plasmid DNA was mixed with competent cells. Electroporation was done in a 1 mm cuvette using a Gene Pulser Xcell (BioRad, München, Germany) with a constant time protocol with input parameters 1.5 kV, 25 μF and 600 Ω. Four hundred μL of recovery solution (Basic Brock medium without pH adjustment) was added and cells were recovered for 30 min shaking at 300 rpm and 75°C. Afterward, 100 μL of cell suspension was plated on plates without uracil and incubated inside a humidity chamber for 6–7 days.

Competent *Escherichia coli* DH5α, Sure2 or ER1821 (NEB) used for cloning and methylation of plasmid DNA, respectively, were grown in at 37°C in LB media supplemented with the appropriate antibiotics.

### Plasmids construction

All plasmids and primers are listed in Supplementary Tables 1, 2.

Different codon optimized *tgp* variants (low and high pKa and a cysteine free version), codon optimized *hfyfp* as well as linker sequences were ordered from GenScript. Genes for fluorescent proteins and linkers were fused using overlap PCR. Resulting fragments were cloned into the listed expression plasmids using *Nco*I and *Apa*I. Promoter sequences were cloned using *Sac*II and *Nco*I. The TGP derived variants YTP (TGP H193Y) and YTP-e (Q65E, H193Y), as well as the monomeric version of hfYFP, mfYFP (hfYFP S147P, L195M, V206K) were created by subsequent site directed point mutagenesis. The codon optimized sequences of *tgp* and *mfyfp* can be found on NCBI under accession numbers PQ130168 and PQ119385, respectively.

If not stated otherwise, genes of interest (*cdvB* and *pcna1*) were amplified from *S. acidocaldarius* DSM 639 gDNA using the primers listed in Supplementary Table 2 and cloned into the expression plasmids (van der Kolk et al., 2020) with *Nco*I and *Xho*I (New England Biolabs GmbH, Frankfurt am Main, Germany). *LacS* was amplified from a plasmid containing the gene pSVA431 (Wagner et al., 2012). Similarly, native promoters were cloned using *Sac*II and *Nco*I (New England Biolabs GmbH, Frankfurt am Main, Germany).

To create FX plasmids for TGP and mfYFP, *in vivo* assembly was used. Primers listed in Supplementary Table 2, containing overlapping sequences of 15–20 bp, were used to amplify both the backbone plasmid (Supplementary Table 1) and the gene encoding the fluorescent protein with the thermolinker. For assembly, 50 ng of linearized plasmid was mixed with varying amounts of the amplified fragment containing the fluorescent protein, followed by transformation into *E. coli*.

Correct plasmids were methylated by transforming them to *E. coli* ER1821 containing pM.EsaBC4I, expressing a methylase (NEB).

### Flow cytometry

Cultures containing the plasmids of interest were grown on liquid Brock media until OD_600_ of 0.3–0.5 and induced with 0.2% xylose for 4 h for TGP containing constructs. For the yellow constructs, induction was done overnight with 0.2% xylose and cells were grown to OD_600_ of 0.3–0.5, as well as for the TGP used for comparison. Samples from expression cultures were taken and kept away from light exposure and warm, when necessary, in a container until injection into a Becton-Dickinson Fortessa flow cytometer equipped with a green laser (488 nm). Filters used for detection were FL12—450/50 and FL13—530/30—550 LP. The number of events recorded per sample was 100,000. Flow cytometry experiments were performed at least three times. Analysis was carried out using FlowJo™ software v10.10. Gating of cells was done manually using the FSC vs SSC plot. The mean fluorescence intensity (MFI) was obtained from the fluorescence histograms of the cell population using FlowJo™. The MFI values were normalized against the MFI of the empty plasmid control. Additionally, MFI values of yellow variants were normalized against TGP MFI values for comparation. The normalized data was then analyzed using GraphPad, where a Welch’s *t*-test was performed to assess the statistical significance of the differences between expression cultures.

### Cell synchronization

Cultures of cells harboring expression plasmids (OD_600_ of 0.1–0.2) were synchronized using acetic acid as described previously (Tarrason Risa et al., 2020). Expression was induced from the beginning of synchronization with 0.1% xylose. Brock medium for washing steps and final resuspension always included xylose. Samples were taken 80–100 min after washing away the acetic acid. For proteasome arrest, 80 min after release, 10 mM bortezomib was added and cells were grown for 30 min at 75°C. Cells were then washed twice with Brock media, placing them back in the incubator for 10 min in between, and incubated again at 75°C. Samples were taken 2–10 min after that.

### Fluorescent microscopy at room temperature and 75°C

For imaging, 3 μL of cell suspension was spotted on an agarose pad (1% agarose in basic Brock medium), air dried, and imaged with an Axio Observer Z1; Zeiss microscope equipped with a Plan-Apochromat 100x 1.40 Oil Ph3 M27 objective. At least three fields were imaged per sample and experiments were repeated three times. Exposure time was of 500 ms-1 s for GFP channel (450–490/500–550) and 500 ms in YFP channel (490–510/520–550).

For thermomicroscopy, we used the precise temperature control unit for optical microscopes VAHEAT (Interherence GmbH, Germany). Therefore 1 mL of culture was taken and added to a VAHEAT substrate chamber, and closed with a small glass slide. The sample was then heated to 75°C for 5 min before imaging. Images were taken with an Axio Observer Z1; Zeiss microscope equipped with a Plan-Apochromat 100x 1.40 Oil DIC M27 objective. The exposure time was 1 s for the GFP- (450–490/500–550) and YFP- (490–510/520–550) channels.

### SDS-page, western blot and in gel fluorescence

Pellets from 10 mL exponentially growing cell cultures (OD_600_ 0.3–0.5) were resuspended in 1x PBS to a theoretical OD of 10. The samples were mixed with SDS-dye (50 mM Tris-HCl pH 6.8, 2% glycerol, 2% DTT, 0.0004% Bromophenol blue, 2% SDS) and boiled, or not, for 10 min at 99°C. Proteins were separated on a 15% sodium dodecyl sulfate-polyacrylamide gel via electrophoresis. Gels were either stained with Coomassie (25% v/v isopropanol, 10% acetic acid, 0.05% Coomassie R) or blotted onto a polyvinylidene fluoride (PVDF) membrane using the semi-wet Western blot Trans-Blot Turbo Transfer System (BioRad). Blocking was done overnight at 4°C using a 0.1% I-Block (Thermo Fisher Scientific) solution in PBS-T (1x PBS + 0.1% Tween 20). The primary antibody α-HA (rabbit) (Cat. No. H6908-100UL, Sigma-Aldrich) was used in a 1:10,000 dilution and incubated for 4 h at 4°C. Primary antibody α-GFP raised in rabbit (cat. No. SAB4301138, Sigma-Aldrich) was used in a 1:10,000 dilution and incubated overnight at 4°C. The membrane was washed 3 times with PBS. The secondary antibody, anti-rabbit coupled to HRP (horseradish peroxidase) (1:10,000 dilution, Cat. No. 65-6120, Invitrogen), was then applied and incubated for 3 h. Chemiluminescence reaction was initiated by adding HRP substrate (Clarity Max Western ECL Substrate, BioRad). Signals for the Western blot and in-gel fluorescence were acquired using the iBright FL1500 imaging system (Invitrogen). For in-gel fluorescence, the gel was imaged immediately after protein separation.

## Data Availability

The original contributions presented in this study are included in this article/Supplementary material, further inquiries can be directed to the corresponding author.
