# Supplementary material for: The use of thermostable fluorescent proteins for live imaging in Sulfolobus acidocaldarius
Source: Front Microbiol. 2024 Sep 9;15:1445186. doi: 10.3389/fmicb.2024.1445186 (PMC11416942; doi:10.3389/fmicb.2024.1445186)
Supplement: Supplementary file 1 [file Data_Sheet_1.pdf]

Supplementary material to

The use of thermostable fluorescent proteins for live imaging in *Sulfolobus acidocaldarius*

Alejandra Recalde, Jasmin Abdul Nabi, Pierre Junker, Chris van der Does, Jana Elsasser, Marleen van Wolferen and Sonja-Verena Albers*

[1] Molecular Biology of Archaea, Institute of Biology - Microbiology, University of Freiburg, 79104 Freiburg, Germany

***Correspondence:**

S.V. Albers, e-mail: sonja.albers@biologie.unifreiburg.de, tel.: +49(0)761 203 2630

Keywords: Sulfolobus, Fluorescent Microscopy, Thermal Fluorescent Protein, Archaea, In vivo localization, Thermomicroscopy

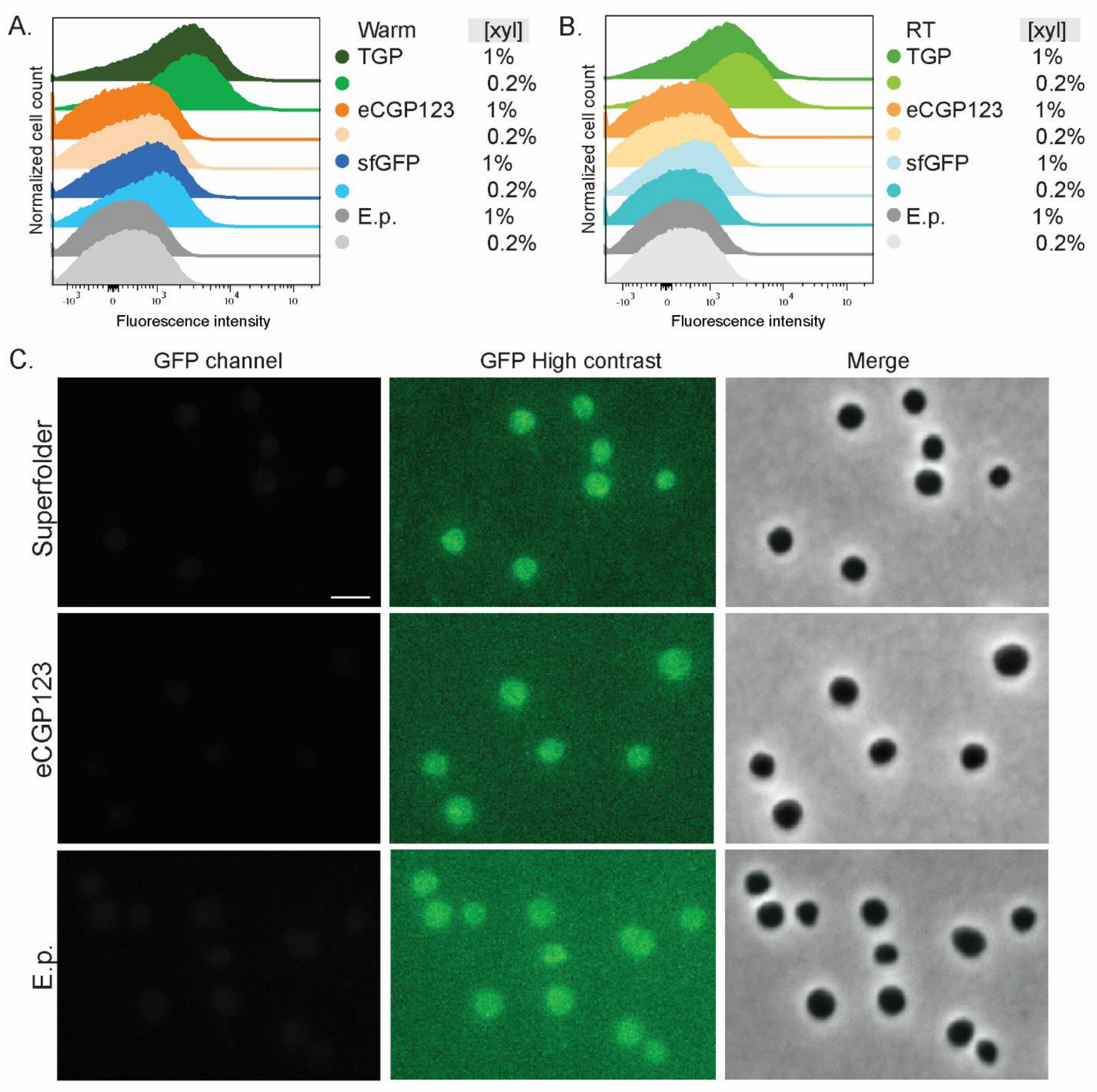

Figure S1. *S. acidocaldarius* expressing different green fluorescent proteins. Fluorescence intensity in flow cytometry of (A) preheated samples (warm) and (B) samples transported at room temperature, both induced with 0.2 or 1% xylose. Number of events for FC: 100.000. (C) Fluorescent microscopy and merge with phase contrast images of cells induced with 0.2% xylose and at room temperature. Contrast settings were the same as for TGP in figure 1C, additionally high contrast was used to show low levels of fluorescence.

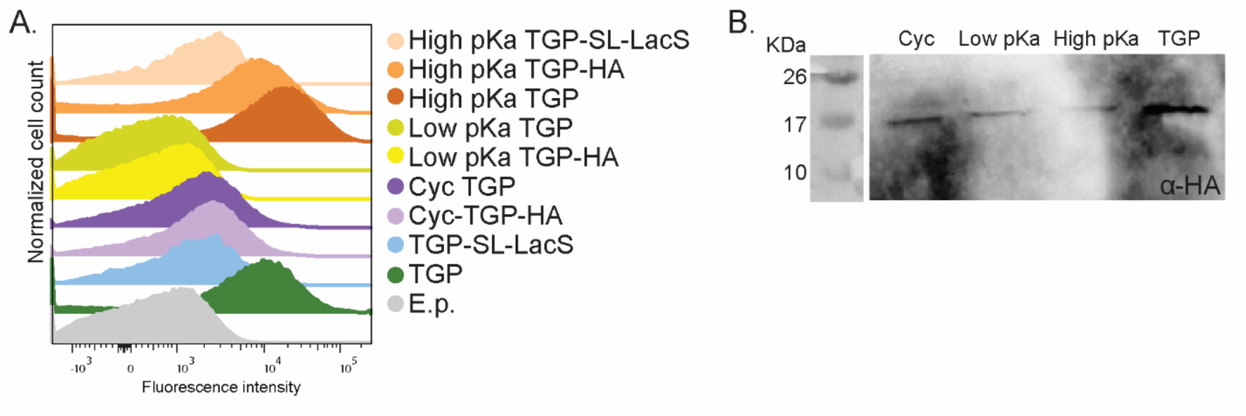

Figure S2. TGP variants with mutations introduced to modify pKa value and sulfur bridges. (A) Fluorescence intensity in flow cytometry at RT and 0.2% xylose induction. Number of events per sample: 100.000. (B) Western blot of the HA tagged versions of the different protein. Samples were normalized to the same theoretical OD_{600}.

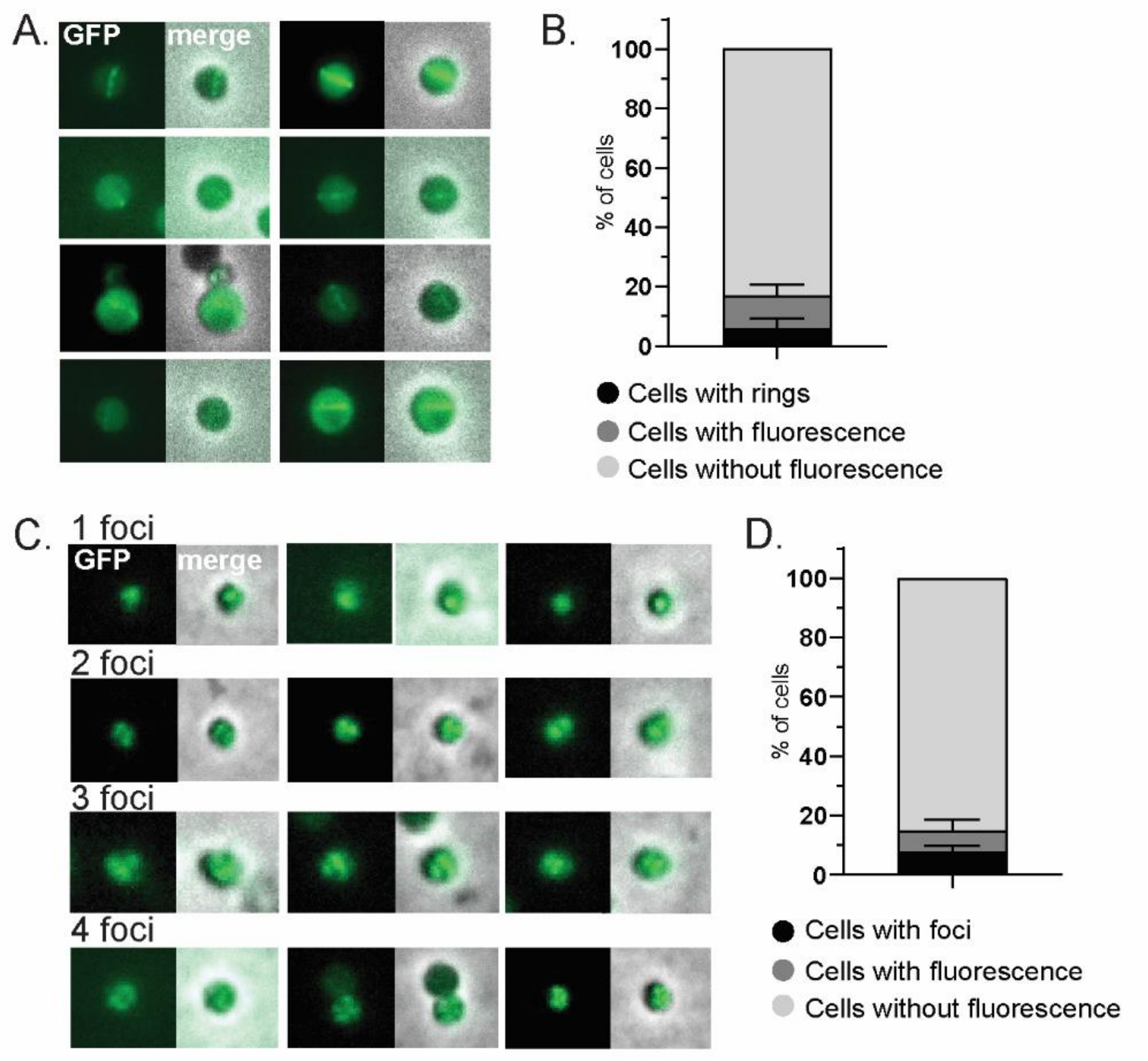

Figure S3. *In vivo* cell localization in *S. acidocaldarius* cells. (A) Examples of TGP-TL-CdvB rings. (B) Percentage of cells that show fluorescence of localization patter (rings) in per image, mean values of triplicates. (C) Sample images of localization foci of TGP-TL-PCNA1. (D) Percentage of cells with localization foci and fluorescence, mean values of triplicates.

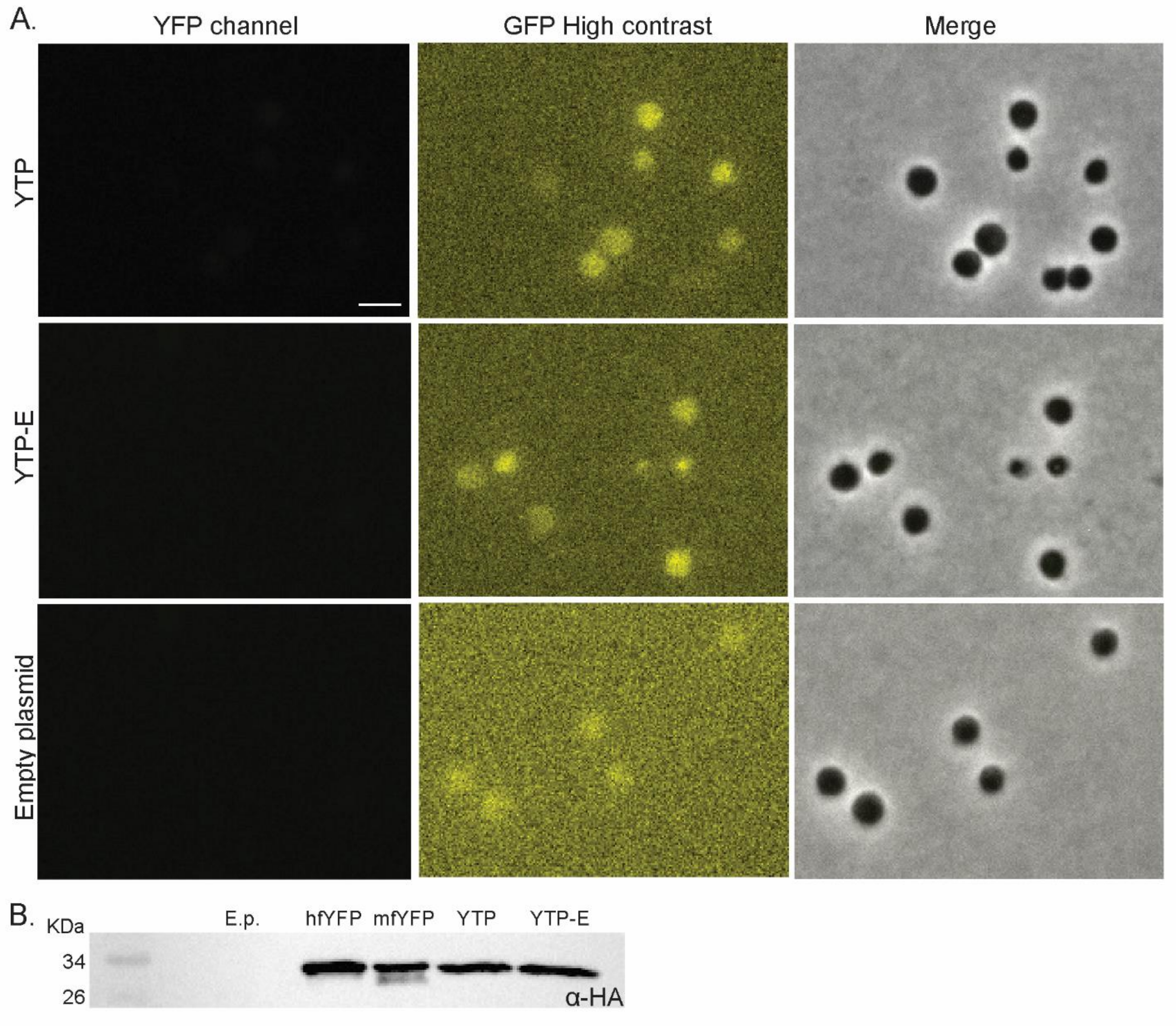

Figure S4. (A) Microscopy images of yellow fluorescent proteins. Fluorescent microscopy, phase contrast and a merged image of cells expressing YTP and YTP-E. Contrast settings were the same as for mfYFP in figure 4, additionally high contrast was used to show low levels of fluorescence. (B) Western blot using anti HA antibodies, showing expression levels of the proteins used for FC and microscopy studies. E.p.: Empty plasmid.

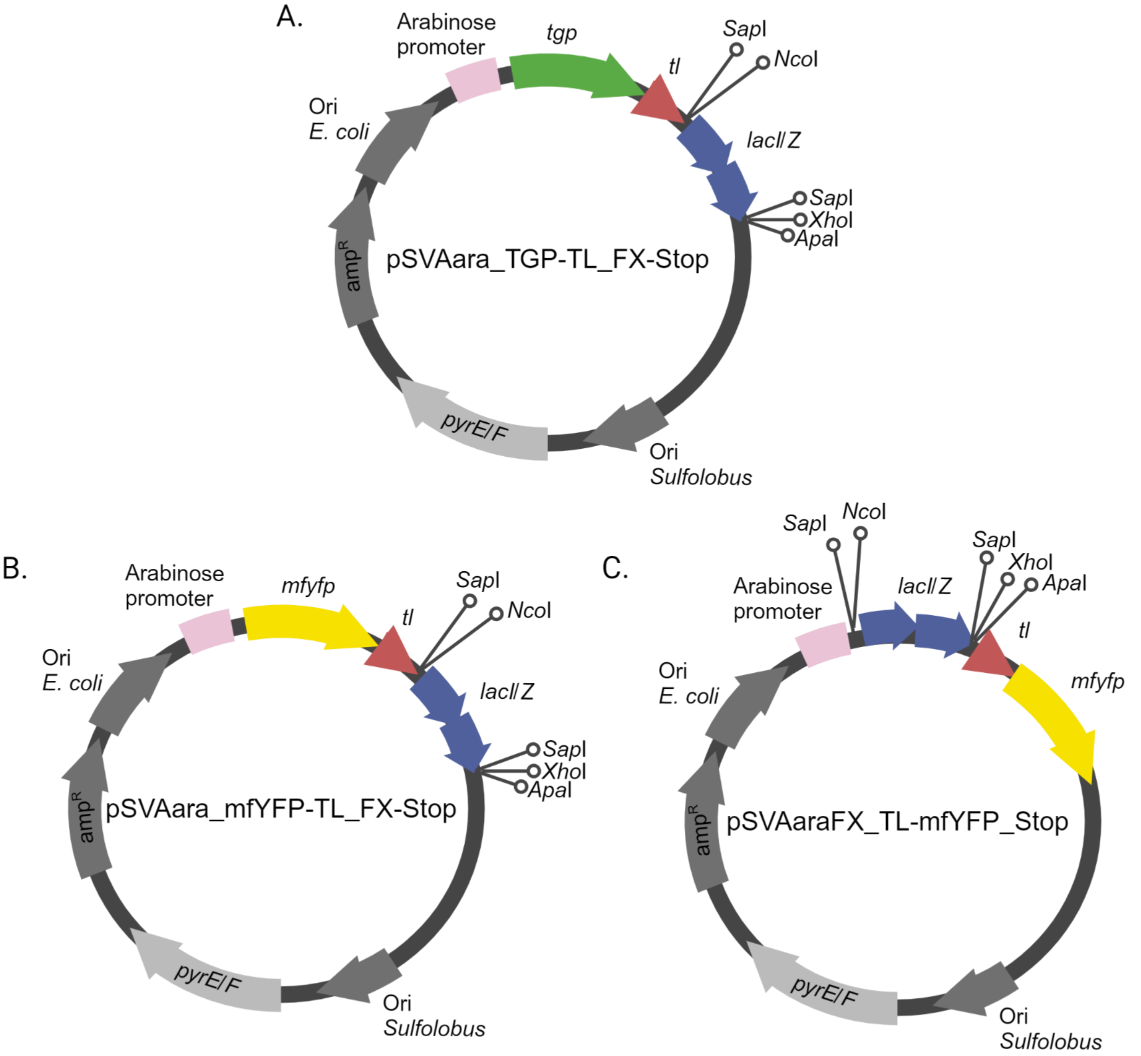

Figure S5. Schematic design of the plasmids (A) pSVAaraTGP-TL_FXStop (pSVA6514), (B) pSVAara_mfYFP-TL_FX-Stop (pSVA7000) and (C) pSVAara_FX_TL-mfYFP-Stop (pSVA7001), containing an arabinose promoter, *tgp* or *mfYFP*, the thermolinker (*tl*) and *lacI/Z* flanked by restriction sites *Nco*I and *Xho*I to insert a gene of interest in frame as TGP-TL-gene, mfYFP-TL-gene or gene-TL-mfYFP, respectively, followed by a stop codon. Alternatively, *Sap*I can be used to clone via FX cloning. Amp^R: ampicillin resistance cassette for selection in *E. coli*, *pyrE/F*: cassette for selection in Sulfolobus (uracil auxotrophy), Ori: origin of replication. Created with BioRender.com.

Table S1. Plasmids used in this work

pSVA	pSVA backbone	Description	Reference
araFx_Stop		Backbone for expression plasmids	(van der Kolk et al., 2020)
araFX_HA		Backbone for expression plasmids	(van der Kolk et al., 2020)
araFX_NtHA		Backbone for expression plasmids	(van der Kolk et al., 2020)
**Green fluorescent protein**			
12825	pSVAaraFx_Stop	TGP	This work
2584	pSVAaraFX_NtHA	HA-TGP	This work
2585	pSVAaraFX_HA	TGP-HA	This work
5597	pSVA2585	LacS-SL-TGP-HA	This work
13120	pSVAaraFX_HA	sfGFP-HA	This work
13127	pSVAaraFX_HA	eCGP123-HA	This work
13157	pSVA12825	TGP-SL-LacS	This work
13186	pSVA2585	TGP-SL-LacS-HA	This work
**Linkers**			
13158	pSVAaraFx_Stop	TGP-TL-LacS	This work
13159	pSVAaraFx_HA	TGP-TL-LacS-HA	This work
13184	pSVA2585	LacS-TL-TGP	This work
13143	pSVAaraFx_HA	pSVAara_FL_TGP_HA	This work
13144	pSVAaraFx_HA	pSVAara_RL _TGP_HA	This work
13145	pSVAaraFx_HA	pSVAara_SRL_TGP_HA_	This work
13146	pSVA13144	LacS-RL-TGP-HA	This work
13148	pSVAaraFx_Stop	LacS-RL-TGP	This work
13151	pSVA13145	LacS-SRL-TGP-HA	This work
13154	pSVAaraFx_Stop	LacS-SRL-TGP	This work
13155	pSVA13143	LacS-FL-TGP-HA	This work
13156	pSVAaraFx_Stop	LacS-FL-TGP	This work
**TGP variants**			
13178	pSVAaraFx_Stop	Cyc TGP (C103S C113S C171S) (codon adapted gene ordered in GenScript)	This work
13179	pSVAaraFx_Stop	Low pKA TGP (K5E K9E K21E K137E R186E K202E) (codon adapted gene ordered in GenScript)	This work
13180	pSVAaraFx_Stop	High pKA TGP (E26R E45R E73R E89R E109R E117R D187R) (codon adapted gene ordered in GenScript)	This work
13181	pSVAaraFX_HA	Cyc TGP-HA	This work
13182	pSVAaraFX_HA	Low pKa TGP-HA	This work
13183	pSVAaraFX_HA	High pKA TGP -HA	This work
13187	pSVA13183	High pKa TGP-SL-LacS-HA	This work
**Cell localization**			

6500	pSVAaraFX_Stop	Expression TGP-TL-cdvB (saci_1373)	This work
6552	pSVAaraFx_Stop	native promoter PCNA1, expression TGP-TL-PCNA1 (saci_0826)	This work
**Yellow fluorescent proteins**			
6506	pSVAaraFX_Stop	hfYFP (codon adapted gene ordered in GenScript)	This work
6545	pSVAaraFX_Stop	mfYFP	This work
6593	pSVAaraFX_HA	mfYFP-HA	This work
6594	pSVAaraFX_NtHA	HA-mfYFP	This work
6586	pSVA12825	YTP	This work
6580	pSVAaraFX_HA	YTP-HA	This work
6581	pSVAaraFX_NtHA	HA-YTP	This work
6576	pSVA6586	YTP-E	This work
6582	pSVAaraFX_HA	YTP-E-HA	This work
6583	pSVAaraFX_NtHA	HA-YTP-E	This work
**Generated plasmids for cloning**			
6514	pSVAaraFX-Stop	pSVAara_TGP-TL_FXStop	This work
7000	pSVAaraFX-Stop	pSVAara_mfYFP-tl_FX-Stop	This work
7001	pSVAaraFX-Stop	pSVAaraFX_tl-mfYFP_Stop	This work

Table S2. Primers used in this study

**Primer No.**	**Sequence (3´->5´)**	**Use**
9355	GCGCCTCGAGTCCACCACCACTACCACC	Rv *XhoI tgp*
9356	GCGCCCATGGCGGCAAGTGTAATAAAGCCTGAGATG	Fw *NcoI tgp*
8662	GACTCCATGGACTCATTTCCAAATAGCTTTAGG	Fw *lacS*
8663	GACTGGATCCGTGCCTTAATGGCTTTACTGGAGGTACGCTAT	Rv *lacS*
11657	ATATATGCTCTTCTAGTTCTAAGGGTGAGGAGTTATTCACTGGA	Fw *Sap*I *sfGFP*
11658	TATATAGCTCTTCATGCCCTTATTTATATAACTCATCCATTCCA	Rv *Sap*I *sfGFP*
11680	ACGTCCATGGATGAGTGTAATAAAGCCTGAAATGA	Fw *Nco*I *eCGP123*
11681	GCATCTCGAGTTTAGCTTGACTTGGTAACATA	Rv *Xho*I *eCGP123*
12026	GACTCCATGGACTCATTTCCAAATAGCTTTAGGTTT	Fw *Nco*I *lacS*
12027	GACTCCATgGGAAATTCACCTGATCCAGCAGCTGAACCAGCTGATCC GTGCCTTAATGGCTTTACTG	Rv *XhoI lacS*
12045	ATGGCGGCAAGTGTAATAAAGCCT	Fw plasmid FX TGP
12046	CTCGAGTGCTGAAGAGCCGGATCC	Rv plasmid FX TGP
12047	GGATCCGGCTCTTCAGCACTCGAG	Fw fragment pUC linkers
12048	AGGCTTTATTACACTTGCCGCCAT	Rv fragment pUC linkers
12058	GCAGCAGCTTCCTTTGCAGCTGCCTCTTTAGCTGCAGCTTCCTCGAGTGC TGAAGAGCCGGAT	Fw rigid linker T4PKN
12059	AAAAGAGGCAGCTGCTAAGGAAGCAGCAGCTAAAATGGCGGCAAGTGTAA TAAAGCCTGA	Rv rigid linker T4PKN
12062	GCATCTCGAGGGATCAGCTGGTTCAGCTGCTGGATCAGGTGAATTTATGGA CTCAT TTCCAAATAGCTTTAGG	Rv *XhoI* l*acS*
12071	CATGCATGGGCCCTTACTCAAGCGATCCACCACCA	Rv *lacS* thermolinker
12073	GCATCTCGAGATGGACTCATTTCCAAATAGCTTTAGG	Fw *XhoI* LacS
12074	GGATCAGCTGGTTCAGCTGCTGGATCAGGTGAATTTATGGACTCATTTCCAA ATAGCTTTAGG	Fw LacS thermolinker
12075	AAATTCACCTGATCCAGCAGCTGAACCAGCTGATCCTCCACCACCACTAC CACCACCAGA	Rv TGP thermolinker
12088	CATGCCATGGGAGCACATGCTTCTGTAA	Fw *Nco*I *tgp*
12092	ATGGCGGCAAGTGTAATAAAGCCT	Fw to put pSVA13184 in frame
12093	AAATTCACCTGATCCAGCAGCTGAA	Rv to put pSVA13184 in frame
13216	ATGCCTCGAGTGCTCCTCCGCCACTTCCTCCACCA	Rv TGP variants
13247	GGATCAGCTGGTTCAGCTGCTGGATCAGGTGAATTTATGTTTGATAAGTTAT CGA TAATT	Fw *cdvB* thermolinker
13248	TGCATCTCGAGACCCTCAAGAACAATTAGACCCTT	Rv *Xho*I *cdvB*
13281	ATGCCCATGGTATCAAAGGGTGAAGAAC	Fw *Nco*I hfYFP/mfYFp
13282	TGCATCTCGAGTTTATATAATTCATTCATATCATGAGTT	Rv *Xho*I hfYFP
13964	CATAATGTTTATATAACAGCAG	Fw point mutation S147P in hfYFP

13965	AGGATTGAAATTATATTCTAACTTATG	Rv point mutation S147P in hfYFP
13966	CCTGATAATCATTATCTAAGTTATCAAT	Fw point mutation L195M in hfYFP
13967	CATTAGTACTGGTCCATCACCTATT	Rv point mutation L195M in hfYFP
13968	AAACTATCTAAGGATCCTAATGAGAAA	Fw point mutation V206K in hfYFP
13969	TGATTGATAACTTAGATAATG	Rv point mutation V206K in hfYFP
13981	GGATCAGCTGGTTCAGCTGCTGGATCAGGTGAATTTATGATAAAAAATA AAGTTATG TCAGAT	Fw thermolinker *pcna1*
13982	TGCATCTCGAGAGATAGTTTGGGTGCAAGTAAATA	Rv *Xho*I *pcna1*
13983	ATCGCCGCGGTACCTTATATCTCTTCGATAGTAC	Fw *Sac*II Native promoter PCNA2
13984	GACTCCATGGGAGGTTTCCCCGTATTATTATT	Rv *Nco*I Native promoter PCNA1
14083	TATGAAGTTGATCATAGAATAG	Fw point mutation H193Y in TGP
14084	AGCATCTGGTAATCTAACATC	Rv point mutation H193Y in TGP
14702	GATAAGCGTCTTACTTATCATAATGGTATCAAAGGGTGAAGAACTATTC	fw mfYFP for pSVA7000
14703	ATTCACCTGATCCAGCAGCTGAACCAGCTGATCCTTTATATAATTCATTCATATCA TG	Rev mfYFP for pSVA7000
14704	GCTGCTGGATCAGGTGAATTTCCATGGCTAGTTGAAGAGC	fw pSVAAraFXStop from pSVA7000
14705	TATGATAAGTAAGACGCTTATCAATTATTAGTATTTATTTTAACATGTTAAACGTTA TAAACGCTGAGTAAT	Rev pSVAAraFXStop from pSVA7000
14706	GGATCAGCTGGTTCAGCTGCTGGATCAGGTGAATTTATGGTATCAAAGGGTGAA GAA	fw mfYFP for pSVA7001
14707	TATAGTGAGACTTGAGAAAGTGGGCCCTTATTTATATAATTCATTCATATCATGAG TTATTCCA	Rev mfYFP for pSVA7001
14708	TAAGGGCCCACTTTCTCAAGTCTCACTATACCAAATGAGTTTTCTTTTAATCTTAT	fw pSVAAraFXStop from pSVA7001

14709	ACCTGATCCAGCAGCTGAACCAGCTGATCCCTCGAGTGCTGAAGAGCCGGAT	rev pSVAAraFXStop from pSVA7001
